# Imaging the effect of the circadian light–dark cycle on the glymphatic system in awake rats

**DOI:** 10.1073/pnas.1914017117

**Published:** 2019-12-17

**Authors:** Xuezhu Cai, Ju Qiao, Praveen Kulkarni, Ian C. Harding, Eno Ebong, Craig F. Ferris

**Affiliations:** ^a^Center for Translational NeuroImaging, Northeastern University, Boston, MA 02115;; ^b^Department of Bioengineering, Northeastern University, Boston, MA 02115;; ^c^Department of Chemical Engineering, Northeastern University, Boston, MA 02115;; ^d^Department of Psychology, Northeastern University, Boston, MA 02115;; ^e^Department of Pharmaceutical Sciences, Northeastern University, Boston, MA 02115

**Keywords:** brain temperature, suprachiasmatic nucleus, substantia nigra, circadian cycle, vascular density

## Abstract

The glymphatic system functions in the removal of potentially harmful metabolites and proteins from the brain. Dynamic, contrast-enhanced MRI was used in fully awake rats to follow the redistribution of intraventricular contrast agent entrained to the light–dark cycle and its hypothetical relationship to the sleep–waking cycle, blood flow, and brain temperature in specific brain areas. Brain areas involved in circadian timing and sleep–wake rhythms showed the lowest redistribution of contrast agent during the light phase or time of inactivity and sleep in rats. Global brain redistribution of contrast agent was heterogeneous. The redistribution was highest along the dorsal cerebrum and lowest in the midbrain/pons and along the ventral surface of the brain. This heterogeneous redistribution of contrast agent paralleled the gradients and regional variations in brain temperatures reported in the literature for awake animals. Three-dimensional quantitative ultrashort time-to-echo contrast-enhanced imaging was used to reconstruct small, medium, and large arteries and veins in the rat brain and revealed areas of lowest redistribution overlapped with this macrovasculature. This study raises new questions and theoretical considerations of the impact of the light–dark cycle, brain temperature, and blood flow on the function of the glymphatic system.

## Significance

Homeostasis and the daily rhythms in brain function and temperature are coupled to the circadian light–dark cycle. MRI was used to study the redistribution of intraventricular contrast agent in awake rats during the night when they are active and during the day when at rest. Redistribution is lowest during the day and highest at night and parallels the gradients and regional variations in brain temperatures reported in the literature. The brain areas of low parenchymal redistribution are associated with high temperatures and have a high density of blood vessels that may be an essential part of the organization of the glymphatic system regulating brain temperature, blood gases, nutrients, metabolites, and waste products over the light–dark cycle.

The glymphatic system, recently discovered in both rodents and humans, plays an essential role in clearing metabolic waste from the brain (1, 2). While the mechanisms contributing to the movement between the cerebral spinal fluid (CSF) and interstitial fluid (ISF) at the level of the neurovascular unit remain a point of debate, whether through convection as described in the glymphatic hypothesis (1) or conventional diffusion and solute transport (3–7), bulk flow or convection of CSF along the perivasculature through deep arteries and arterioles is a key feature in the movement and clearance of waste from the brain (1, 8, 9). Due to its function in waste clearance, there is a growing literature that the glymphatic system may play a critical role in the etiology and pathophysiology of several CNS disorders like Alzheimer’s and Parkinson’s diseases (1, 10).

Recent studies in animals and humans report perivascular movement is affected by the sleep–wake cycle, with clearance of waste occurring during the diurnal period associated with rest and sleep (11–15). Sleep facilitates ISF efflux and the clearance of metabolites from the brain (11). Lactate, a by-product of anaerobic glycolysis from enhanced metabolic activity, accumulates during the wake stage of the sleep–wake cycle and declines during sleep (12), a function ascribed to the glymphatic system. Several studies report the clearance of amyloid beta and tau proteins through the glymphatic system is enhanced with sleep (13–15). In a recent study, Holth and colleagues (15) showed sleep deprivation in mice elevated tau protein levels in the brain. However, most living organisms exhibit fluctuations in physiological processes such as heart rate, blood pressure, body temperature, and hormone level as well as behavioral and cognitive functions in the context of the light–dark cycle despite the state of arousal (16, 17). These rhythms are fundamentally regulated by the entrainment of the suprachiasmatic nucleus in the hypothalamus to the light–dark cycle. The relationship of glymphatic clearance to the light–dark cycle has not been fully addressed. Here we present data based on the light–dark cycle that suggest the influx and parenchymal distribution of intraventricular contrast agent are lowest during the diurnal cycle associated with rest and sleep, a finding consistent with previous publications on glymphatic function. However, this study looks at redistribution of contrast agent in specific brain areas in awake animals and the association of these areas with the density of blood vessels. The data raise new theoretical considerations of the potential importance of brain temperature and blood flow for the function of the glymphatic system.

## Materials and Methods

### Animals.

Twelve Sprague–Dawley male rats (250 to 300 g) were purchased from Charles River Laboratories, with six housed on a 12-h:12-h light–dark cycle (lights on at 7:00 AM) and six housed on a reversed 12-h:12-h light–dark cycle (lights on at 7:00 PM) for a period of 2 wk. An additional rat was ordered to assess blood vessel distribution. Animals were maintained in ambient temperature (22 to 24 °C) and provided with food and water ad libitum. All animals were cared for in accordance with the NIH *Guide for the Care and Use of Laboratory Animals* (18). Methods and procedures used in this study were preapproved by the Northeastern University Institutional Animal Care and Use Committee (IACUC).

### Acclimation for Awake Imaging.

The use of anesthesia to study the glymphatic system has been a subject of some debate following the publication by Gakuba et al. (19) showing data that anesthesia impairs flow. However, a detailed study testing several anesthetic formulae combined with electroencephalography (EEG) and cardiorespiratory measures reported a mixed effect on movement, with some anesthetics like ketamine/xylazine enhancing movement while high-dose isoflurane reduced movement (20). To avoid the confound of anesthesia, perivascular movement was evaluated in fully awake rats. To prepare rats for awake imaging, they underwent daily acclimation over 5 consecutive days. Rats on the reversed light–dark cycle were acclimated under red-light illumination. Rats were lightly anesthetized with isoflurane and placed into a copy of the restraining system used during awake imaging. When fully conscious, the animals were placed into a dark mock scanner tube with a sound recording of a standard MRI pulse sequence playing in the background. This acclimation procedure has been shown to significantly reduce plasma CORT, respiration, heart rate, and motor movements when compared with the first day of acclimation. The reduction in autonomic and somatic response measures of arousal and stress improves the signal resolution and MR image quality (21).

### Surgical Procedure.

Just prior to imaging, rats were anesthetized with 2 to 3% isoflurane and received an s.c. injection of the analgesic Metacam (meloxicam, 5 mg/mL solution) at a dose of 1 mg/kg. The scalp was incised and a burr hole was made in the skull for implantation of sterile PE10 tubing (Braintree Scientific) aimed at the right lateral cerebroventricle using the stereotaxic coordinates 1.0 mm posterior to the bregma, 2.0 mm lateral to the midline, and 4.0 mm in depth from dura. The tubing, ca 60 cm in length and prefilled with gadobenate dimeglumine (MultiHance) 1.06-kDa contrast agent (CA) diluted 1:20, was fixed in place with cyanoacrylic cement and connected to a 0.3-mL syringe needle filled with the contrast agent that could be positioned just outside the bore of the magnet. This injection method has been used in previous studies to deliver drugs directly to the brain during awake imaging (22, 23). The surgery on rats maintained on the reversed light–dark cycle was performed under red-light illumination.

### Imaging Acquisition.

Rats were imaged within the first 4 h of the onset of the light–dark cycle. The room housing the magnet was kept in the dark for the entire scanning period for rats maintained on the reversed light–dark cycle. MRI was performed on a Bruker BioSpec 7-T/20-cm USR MRI spectrometer controlled by ParaVision 6.0 software. Radio frequency signals were sent and received with a custom quadrature volume coil built into the animal restrainer (Animal Imaging Research). Immediately after surgery, rats were quickly placed into the head coil and restraining system, a procedure that takes less than a minute (https://www.youtube.com/watch?v=JQX1wgOV3K4). The design of the restraining system includes a padded head support obviating the need for ear bars helping to reduce animal discomfort while minimizing motion artifact. The T1-weighted images were collected using a fast low angle shot sequence. The imaging parameters included a time to repeat/time to echo of 300 ms/2.5 ms and flip angle (FA) of 30°. With a data matrix of 20 × 256 × 256 and a field of view of 16 × 30 × 30 mm, the size of each voxel was 0.8 × 0.117 × 0.117 mm. An area as small as the suprachiasmatic nucleus was calculated to have ca 21 voxels occupying a volume of 0.22 mm^3^. Preinjection scans in both axial and sagittal views were collected, followed by contrast agent administration. A total volume of 10 µL of contrast agent was injected into the lateral ventricle at a rate of 1.6 µL/min using a syringe pump (Harvard Apparatus 22). This rate of injection is reported to keep intracranial pressure within a normal range (24). Sequential T1-weighted images were collected every 2 min and 41 s for 2 h after contrast administration. A contrast agent phantom was attached to the head coil during each scan for validation of image intensity rescaling. Physiological parameters including respiratory rate and oxygen saturation were monitored during imaging sessions. The degree of head motion and displacement was determined in the six rats imaged during the light phase to assess their level of arousal and compared with an equal number of randomly selected Sprague–Dawley rats from a previous study imaged while anesthetized with isoflurane (*SI Appendix*, Fig. S1). Motion parameters X, Y, Z direction displacement as well as the total Euclidean distance of X, Y, Z from motion correction preprocessing were used for the statistical analysis.

### Blood Vessel Distribution.

To evaluate blood vessel distribution across the whole brain, three-dimensional (3D) quantitative ultrashort time-to-echo contrast-enhanced (QUTE-CE) MRI was performed using ferumoxytol, a superparamagnetic iron oxide nanoparticle contrast agent (25, 26). The rat was anesthetized and given a tail vein injection of heparinized saline followed by a volume of ferumoxytol calculated to produce a concentration of 200 µg/mL Fe in the blood immediately after administration. QUTE-CE scans were performed before and after contrast agent administration. The UTE pulse sequence used two 200-kHz fixed trajectories with acquisition parameters of TR 4 ms, TE 0.01 ms, and FA 20°. The field of view was 3 × 3 × 3 cm^3^ with a matrix size of 180 × 180 × 180. Images were averaged over two scans.

### Imaging Analysis.

Image preprocessing and analysis were performed with a combination of 3D slicer, AFNI, FSL, MIVA, and MATLAB software. Reconstructed T1-weighted image data were rescaled to the original intensity measurements (divided by the receiver gain and multiplied by the scaling factor called SLOPE) for each time point for each subject. The signal intensities extracted from manually drawn regions of interest (ROIs) in the CA phantom at different time points were used for validation (*SI Appendix*, Fig. S2). Head motion correction was conducted for the first sequential acquisition session with the middle volume as the reference image using the AFNI program. MR images were skull-stripped semiautomatically by applying a manually drawn brain mask of the reference image to coregistered 4D concatenated anatomical data. Spatial smoothing with FWHM of 0.2 mm was performed on all images for each rat.

**Fig. 1.**
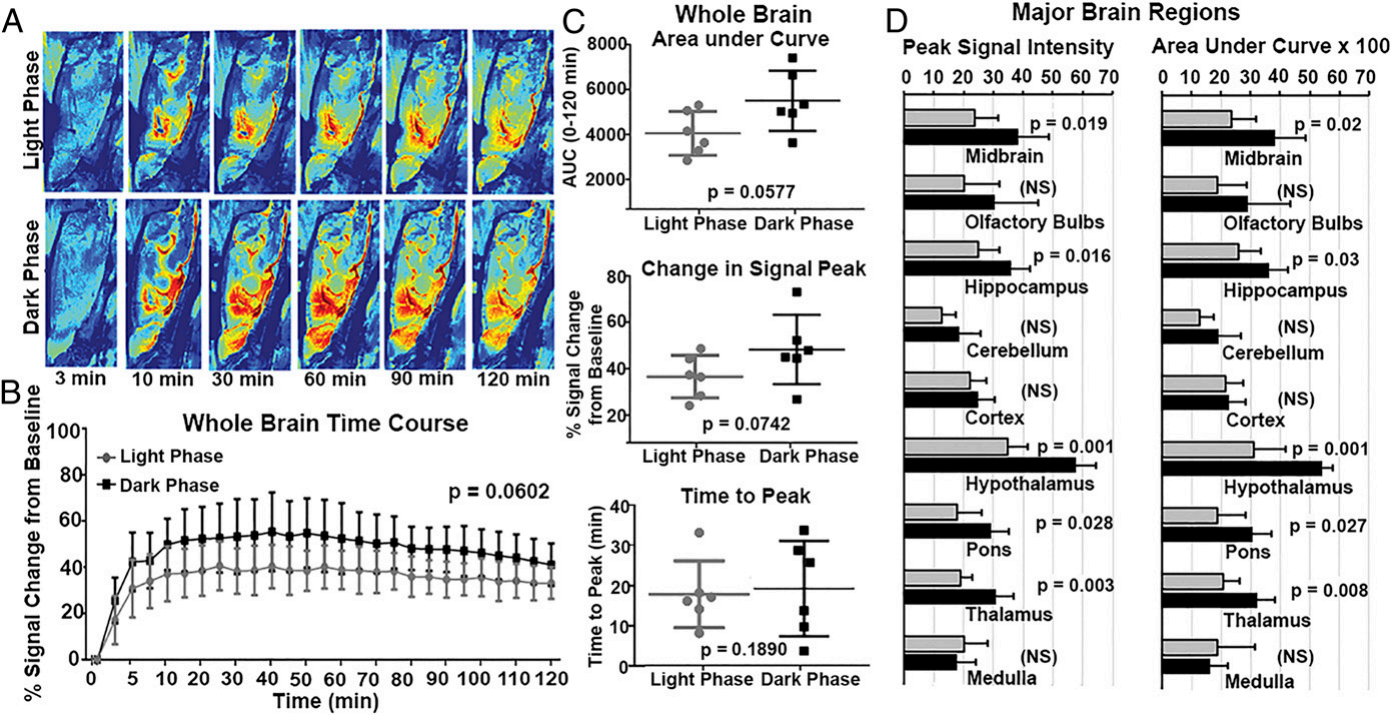
Brainwide parenchymal kinetics of contrast agent in awake rats. (*A*) Examples of heat maps of contrast agent dynamics along the perivascular pathway and into the parenchyma over 2 h (i.e., 3, 10, 30, 60, 90, and 120 min) in both experimental groups (sagittal view). (*B*) Time-to-signal curve over 2 h reflecting the redistribution of contrast agent in the whole-brain area post injection (*n* = 6 light phase; *n* = 6 dark phase). Two-way ANOVA was performed followed by Sidak multiple correction. There is a trend toward significance between the light and dark phases (*P* = 0.0602). (*C*) Comparison of parameters (i.e., area under the curve, signal peak, and time to peak) acquired from exponentially fitted time-to-signal curves in the two experimental groups. Unpaired *t* tests were performed with *P* < 0.05 considered as a significant difference. (*D*) Comparisons of peak signal and area under the curve in major brain regions (midbrain, olfactory bulbs, hippocampus, cerebellum, cortex, hypothalamus, pons, thalamus, and medulla) in the two experimental groups. Error bars represent mean ± SD. NS, not significant.

A 3D MRI rat brain atlas (Ekam Solutions) containing 173 brain regions was used for brain segmentation for each subject by manually registering the atlas to the subject space using a rigid registration method. The ROIs for the aqueduct and pituitary recess were drawn to assess contrast agent kinetics in the ventricular conduits. The calculations for whole-brain analysis shown in Fig. 1 were done after masking the contribution of the ventricular system. Glymphatic redistribution of intraventricular CA was evaluated by the percentage change of signal intensity as a function of time after contrast administration using the following equation: ([signal intensity − baseline intensity]/[baseline intensity × 100]). The time-to-signal curves were fitted with two-component exponential functions. Area under the curve (AUC) over 60 and 120 min, peak signal intensity, and time-to-peak signal intensity were extracted from the curves for further statistical analysis. Data from signal peak and time to peak were extracted from curves fitted to the raw time-to-signal data with two-compartment exponential functions:

$$y=ae^{bx}+ce^{dx}.$$

Quality assurance was performed for awake imaging, and image volumes with excessive motion or artifacts during the scanning session were excluded from analysis. These data points were constructed with predicted values from fitting curves plus or minus random noise for statistical analysis.

**Fig. 2.**
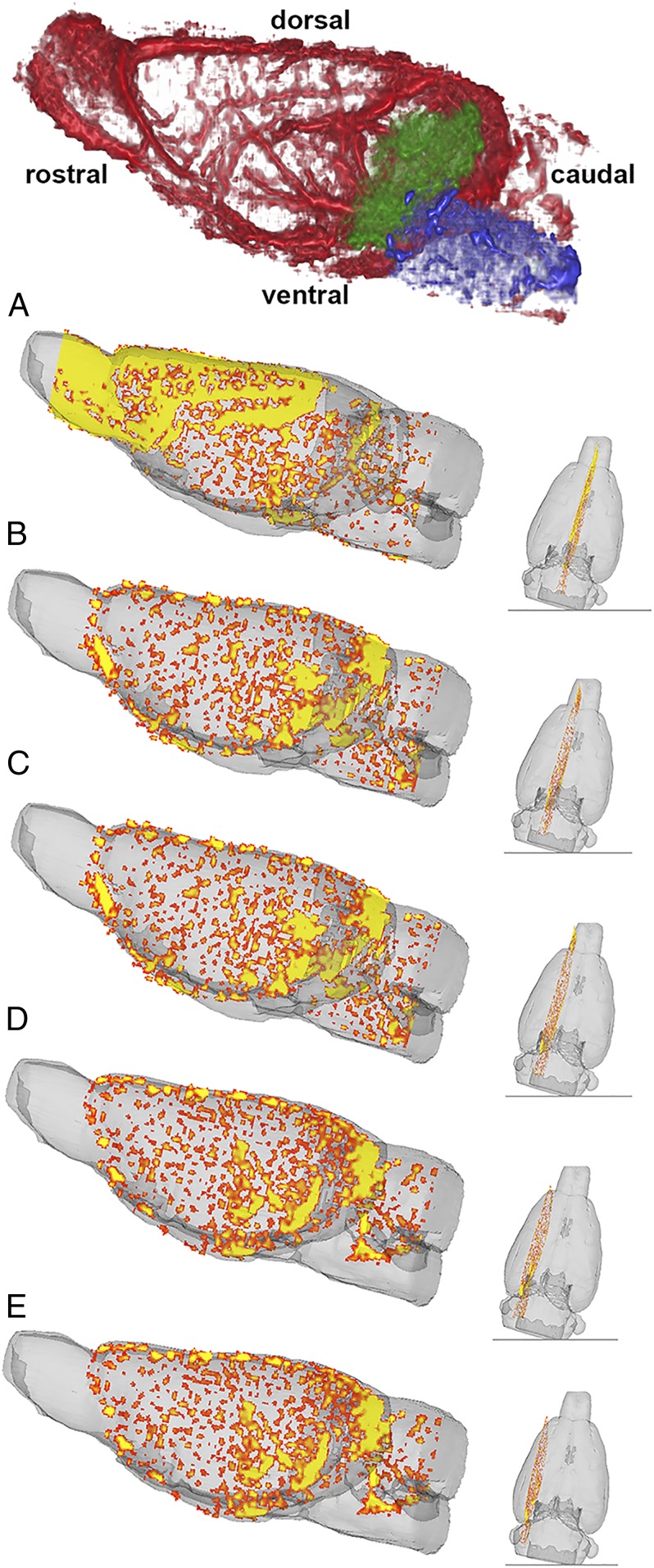
Whole-brain QUTE-CE 3D vascular density map. Whole-brain vascular density map is shown (*Top*) (red) with midbrain vasculature colored in green and the vasculature in the pons colored in blue. Sagittal sections (*A*–*E*, *Left*) from the vascular density map after thresholding show the macrovasculature with the largest arteries and veins colored in yellow and smaller arteries and veins colored in orange. The coronal sections (*A*–*E*, *Right*) show the lateral position of the larger sagittal section.

The precontrast and postcontrast UTE images were aligned using the SPM12 toolbox (https://www.fil.ion.ucl.ac.uk/spm/). The vascular density was calculated from UTE images using $f_{B}=\frac{I_{1}-I_{0}}{I_{B1}-I_{B0}}$ as described in refs. 25 and 26. $I_{B1}$ is the average signal intensity of the blood region in the postcontrast image and is measured by drawing an ROI in the superior sagittal sinus in 3D Slicer. $I_{B0}$ is the average signal intensity of blood in the superior sagittal sinus in the precontrast image. $I_{1}$ is the signal intensity for each voxel in the postcontrast image, and $I_{0}$ is the signal intensity for each voxel in the precontrast image. The 3D MRI rat atlas was used to segment the brain from the whole-head images and to segment different brain regions. The atlas was manually registered to the rat brain image using a rigid registration method. A vascular density image of the whole brain is presented in Fig. 2, *Top* and was created in 3D Slicer. Thresholding was applied to the vascular density map to get vasculature that reflected small, medium, and large arteries and veins, essentially omitting the microvasculature as described previously (26).

### Statistical Significance.

**Fig. 3.**
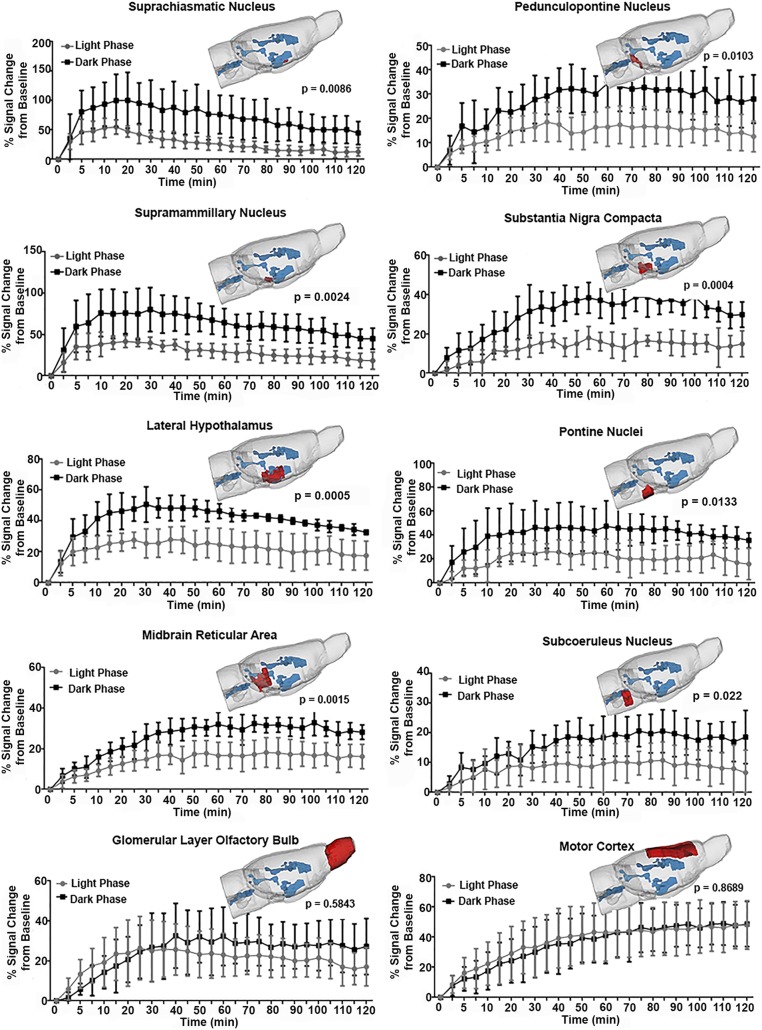
Comparisons of time-to-signal curves in specific brain regions in awake rats in light and dark phases. Signal-to-time curves are shown in specific brain regions including the suprachiasmatic nucleus, pedunculopontine nucleus, supramammillary nucleus, substantia nigra compacta, lateral hippocampus, pontine nucleus, midbrain reticular area, subcoeruleus nucleus, glomerular layer olfactory bulb, and motor cortex. Spatial localizations of these brain regions are shown in the 3D rendering of the rat brain model in red. Ventricular conduits are shown in blue. Two-way ANOVA tests were performed followed by Sidak multiple correction. *P* < 0.05 is regarded as statistically significant in the main effect of the light–dark cycle. Error bars represent mean ± SD.

Statistical analyses were performed using Prism 6 (GraphPad). Raw data points are presented as mean ± SD and two-way ANOVAs were used for comparison with time points being a within-subject factor and circadian cycle being a between-subject factor, followed by a Sidak multiple correction. The parameters extracted from the fitting curves were compared using a two-sample *t* test. *P* < 0.05 was considered statistically significant. Specifically, in Fig. 1 the peak signal and AUC were acquired from the fitting curves based on the original data points and compared using a *t* test. The statistics for Fig. 3 were done using the data points from the preprocessed image. Missing data points or data of insufficient quality were replaced by values from the fitting curve with random noise.

### Data Availability.

The data in *SI Appendix* can be accessed through the following link: http://dx.doi.org/10.17632/2nmnjx66w7.1

## Results

**Fig. 4.**
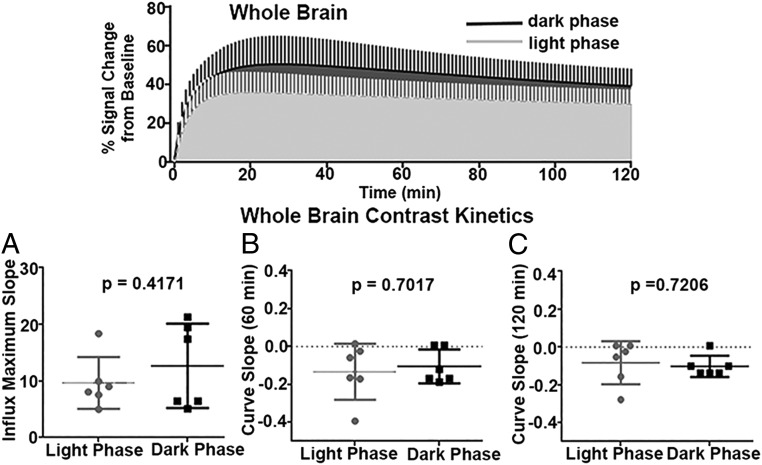
Whole-brain kinetics of contrast agent. (*Top*) Exponentially fitted time-to-signal curves for light and dark phases. (*A*–*C*) Scatterplots comparing three parameters delineating the curve shapes between the two groups. (*A*) Maximum slope of the rising curve. (*B*) Slope of the curve at 60 min. (*C*) Slope of the curve at 120 min. Two-sample *t* tests were performed with *P* < 0.05 as statistically significant. No significant differences were observed among these three parameters in rats in the light and dark phases.

Fig. 1*A* shows a time series of color-enhanced sagittal sections depicting the intensity and distribution of CA following injection into the lateral cerebroventricle. These are representative examples from a rat imaged during the light phase of their circadian cycle and another rat during the night phase of their cycle. T1-weighted images were collected prior to and at 5-min intervals over a 2-h scanning session. There was a trend toward significance (*P* = 0.06) between the average change in signal intensity from the entire brain minus the ventricular system for rats maintained in the light (*n* = 6) versus dark (*n* = 6), shown in Fig. 1*B*. The scatterplots shown in Fig. 1*C*, *Right* show the AUC, change in signal peak, and time to peak for the whole brain from each experimental group. There is no significant difference in time to peak between experimental groups, suggesting the kinetics of redistribution of CA around the brain following injection into the ventricular system is comparable between the light and dark cycles (Fig. 4). Again, there is a trend toward significance between rats imaged in their dark phase as compared with their light phase for AUC (*P* = 0.057) and peak signal intensity (*P* = 0.074) of contrast agent. Shown in Fig. 1*D* are bar graphs for peak signal intensity and AUC for major brain areas over the light–dark cycle. The redistribution of CA was not homogeneous across the brain. While the trend indicated lower redistribution in the light phase than the dark phase, the major brain areas that showed significant differences were the midbrain, hippocampus, hypothalamus, pons, and thalamus.

The mean measures and SDs for respiration (106 ± 38 vs. 104 ± 32) and heart rate (288 ± 95 vs. 379 ± 108) were not significantly different between rats imaged during the light vs. dark phases, respectively (*SI Appendix*, Fig. S3). Conversely, there were significant differences in head motion and direction between rats imaged during the light phase and rats imaged under isoflurane anesthesia (*SI Appendix*, Fig. S1).

Shown in Fig. 4 are the kinetics of contrast agent movement over the whole brain minus the ventricular system for the first 2 h after injection. The time plots above show average curves for the light and dark phases for the raw time-to-signal data using a 2-compartment exponential fitting algorithm. The scatterplots below report the slope of the influx to the maximum change (Fig. 4*A*), the slope of the curve at 60 min (Fig. 4*B*), and the slope of the curve at 120 min (Fig. 4*C*). There were no significant differences in any of these measures.

The redistribution of CA was heterogeneous across the rostral–caudal and dorsal–ventral axes of the brain. Specific brain areas, particularly those in the midbrain/pons and along the ventral surface of the brain, have significantly less redistribution of CA across the light–dark cycle, as shown in Fig. 3. The locations of the areas highlighted (e.g., suprachiasmatic nucleus, supramammillary nucleus, midbrain reticular area, substantia nigra compacta, pontine nuclei, pedunculopontine nucleus, lateral hypothalamus, and subcoeruleus) are shown in red in the 3D glass brain together with their time course of change in signal intensity from baseline to 120 min post injection. The area in blue in the glass brain is the cerebroventricular system. The significance between light and dark phases is denoted by the *P* values. Note the two examples at the bottom, the glomerular layer of the olfactory bulb and the motor cortex, are not significantly different in the redistribution of CA across the light–dark cycle. The motor cortex is an example of all dorsal cerebral areas. Several other brain areas and their parameters derived from regional fitted time–signal curves are provided in *SI Appendix*, Table S1.

Fig. 2 shows sagittal 3D sections of the vascular density in the rat brain. The top figure highlights the entire vasculature (e.g., microvasculature comprising arterioles, capillaries, and venules, and macrovasculature comprising small, medium, and large arteries and veins). The green and blue depict the location of vasculature in the midbrain and pons, respectively. The vasculature to the rest of the brain is shown in red. Sagittal sections shown below depict the location of only the macrovasculature, with the largest arteries and veins shown in yellow and the smaller arteries and veins shown in orange. The smaller coronal sections to the right depict the lateral position of the larger sagittal section, with the midsagittal section shown in Fig. 2*A* and the most lateral section shown in Fig. 2*E*. Note the high density of macrovasculature in the midbrain/pons.

## Discussion

The present study demonstrates that the redistribution of injected intraventricular CA in the glymphatic system can be evaluated in awake rats using dynamic contrast-enhanced MRI. The influx and parenchymal distribution of contrast agent were lowest during the light phase and highest during the dark phase between groups of rats housed under normal and reversed 12-h:12-h light–dark cycles. While these findings are consistent with the literature, the experimental methods and analyses add additional information on the redistribution of intraventricular CA in specific brain regions and potential mechanisms contributing to the diurnal movement of CSF and ISF.

Most of the studies looking at movement of tracers through CSF and ISF have used the intrathecal route afforded by the cisterna magna. In this study, CA was injected into the lateral cerebroventricle at a flow rate of 1.6 µL/min, less than the flow rate of ca 3.0 µL/min reported through ventricles (27) but consistent with production of CSF in rats (28) and in a total volume of 10 µL or 3% of the estimated total CSF volume in the rat brain (28). The formation of CSF comes from the circumventricular organs lining the ventricular system and brain parenchyma (9). This continuous formation together with the compression of the ventricles during respiration and cardiac pulsation moves CSF through the third and fourth ventricles to the subarachnoid space (3). This motion in an anterior-to-posterior direction helps in the transport of endogenous chemical signals, for example, vasopressin and oxytocin released from the hypothalamus (29, 30) and melatonin released from the pineal gland (31). Hence, the injection of CA into the lateral cerebroventricle is a logical choice. The CSF is in communication with the ISF of the brain parenchyma via the perivascular system that provides for CSF–ISF exchange of molecules throughout the extracellular fluid compartment (1, 9). This ventricular route of administration reported by Iliff and colleagues (1) was found to be ineffective in delivering molecules to the brain as injection of fluorescent probes into the lateral cerebroventricles of anesthetized mice showed little to no flux anywhere outside the site of injection. However, intrathecal injection into the cisterna magna revealed the presence of fluorescent signal over much of the brain, leading them and others to adopt this method of delivery for all future studies. It should be noted that the intrathecal route of administration has a decided advantage over injection into the ventricular system because it does not require craniotomy and can be used in the clinic.

To date, there have been several preclinical studies using MRI to follow the movement of injected CA throughout the perivascular system (6, 27, 32–35). The first by Iliff and colleagues (34) injected gadolinium DTPA (molecular mass 938 Da) into the subarachnoid space through the cisterna magna in supine rats anesthetized with isoflurane. In comparison with the present study, the kinetics and distribution were very different, as much of the brain parenchyma did not show influx of CA, and time to peak was ca 90 min in major brain areas like the olfactory bulbs and cerebellum. We present significant accumulation from multiple areas of the brain with a time to peak of ca 30 min. These differences could be due to either the use of anesthesia, direct injection into the subarachnoid space, or the position (35) of the rat during the scanning session. The size of the CAs could not account for the differences or the rate of infusion, as they were both similar. The total amount of CA delivered to the brain by Iliff et al. was much greater and extended over a greater period; nonetheless, influx into the parenchyma was less. In all likelihood, the awake preparation versus anesthesia was the reason for these disparate results. A recently published review by Matsumae and colleagues (36) summarizes the many studies using MRI in humans to characterize the movement of CSF in the brain. The movement of CSF is not circulatory but complex and bidirectional, accelerating and decelerating, and driven by cardiac and respiratory cycles. CSF movement is lowest and even “stagnant” at times along the convexity of the cerebrum. CSF acceleration is highest between the third and fourth ventricles and along the ventral surface of the brain.

The sun is the source of energy that sustains essentially all life on our planet. The solar cycle is the “Zeitgeber” or environmental cue that entrains biological systems to this flow of energy. The suprachiasmatic nucleus (SCN) senses light through the retinohypothalamic tract (37, 38) and entrains to the 24-h light–dark cycle. The SCN is the master clock by which all other circadian rhythms set their clock [for a review, see Honma (39)]. The body has multiple clocks that are coupled and synchronized to maintain homeostasis over the light–dark cycle. How many are involved in the diurnal pattern of glymphatic clearance and to what extent are unknown. The sleep–wake cycle is one notable participant. In a recent study, Holth and coworkers (15) reported levels of tau protein in the ISF of the hippocampus were coupled to the sleep–wake cycle. Levels of tau and lactate from neuronal activity are highest during the dark phase when mice are most active and lowest during the light phase when mice are at rest or sleeping. Six hours of sleep deprivation from manual stimulation or blocking neuronal activity in the hippocampus with tetrodotoxin disrupts this diurnal cycle. Chemogenetic activation of the glutamatergic supramammillary neurons can sustain wakefulness and increase levels of tau and lactate in the hippocampus during the light phase. It is evident that ISF clearance is coupled to the sleep–wake cycle as shown in this and other studies (13, 14); however, the level of physical activity is a confound when interpreting our data. Arousal is undoubtedly associated with the imaging procedure as we show with measures of motion, and while the imaging was only for 3 h during the light phase, this may have increased the redistribution of CA. In other words, without the arousal in the light phase the differences in redistribution of CA between the light–dark cycle may have been greater. Nonetheless, we show clear diurnal differences in CA levels across many specific brain areas. How much of our effect is due to entrainment to the light–dark cycle and to what extent is the diurnal pattern of glymphatic clearance coupled to the sleep–wake cycle and activity? Kervezee et al. (40) reported diurnal variations in P glycoprotein-dependent movement of drug in awake rats. The flux between parenchyma and plasma was highest during the dark phase while CSF clearance was greatest during the light phase. Their findings indicate P-glycoprotein activity is entrained to the light–dark cycle but they note that the function of this protein and the variations in CSF flow over the 24-h period may be affected by sleep–wake activity.

Analyses of many of the 173 specific brain regions showed the SCN to have a significant difference in redistribution of CA over the light–dark cycle. The significant diurnal redistribution of CA was also seen in brain regions that are involved in circadian rhythms of sleep–wake and activity. The pedunculopontine nucleus (PPN) located in the rostral brainstem is part of the ascending reticular activating system with extensive efferent connections to the basal ganglia, thalamus, and lateral hypothalamus (41). The PPN is involved in arousal, motivation, and the organization of rapid eye movement (REM) sleep (42). The PPN has strong connections to the substantia nigra and has been implicated in REM sleep disorders associated with Parkinson’s disease (43). The subcoeruleus nucleus in the rostral brainstem has also been implicated in the control of REM sleep in Parkinson’s (44). The supramammillary nucleus, as noted above, is involved in arousal and a key brain area involved in the sleep–wake cycle (45), as are the lateral hypothalamus (46) and the dorsal raphe (47) (*SI Appendix*, Table S1). All of these areas show significantly less redistribution of CA during the light phase of the circadian cycle. Circadian biology is the biology of anticipation—preparing for what will come. This raises an interesting question—does the enhanced putative clearance from these many areas as suggested in this study during the light phase create the precondition for the next cycle by resetting the chemistry of the external milieu?

It was of interest to see that several brain regions found in the ventral midbrain/pons (e.g., pontine nuclei, pedunculopontine nucleus, supramammillary nucleus, substantia nigra compacta, midbrain reticular area, and periaqueductal gray) showed low redistribution of CA in the light phase. This ventral area of reduced influx and parenchymal distribution of CA was noted in an earlier study by Szentistványi et al. (5) looking at the flux of radiolabeled probes following intrathecal injection in rats. They reported that clearance was five times higher in the midbrain than striatum or white matter tracts. This could be explained by the organization of the arterial circulation in this area. The basilar artery sends a “palisade” of medullary, pontine, and mesencephalic arteries with lateral branches along their length that terminate at the floor of the fourth ventricle and cerebral aqueduct (48). At the junction between pons and midbrain are the pontine and interpeduncular cisterns. These and other subarachnoid cisterns are considered sinks of CSF mixing and high convection for clearance. Bedussi and coworkers (49) provide evidence in rats of a continuous single compartment between the blood vessels in the subarachnoid space, cisterns, and the ventricular system for the movement of CSF. The movement of CSF is most prominent along the ventral surface of the brain up to the olfactory bulbs and provides for clearance through the cribriform plate and nasal lymphatics to cervical lymph nodes (50–52). This ventral brain area has perivascular connections to the olfactory bulb as shown by the rapid convection of intranasal fluorescently labeled tracers (53).

The reduced redistribution of intraventricular CA in the midbrain and ventral surface of the brain due to organization of the blood vessels and cisterns may be an evolutionary coincidence benefiting the neurovascular unit in these brain regions or something that evolved over time to assure homeostasis in metabolically vulnerable areas. While the clearance of metabolites and waste has been the focus of ISF circulation in the brain for all of the studies cited thus far, not to be forgotten is the role blood circulation plays in helping to regulate brain temperature. The brain utilizes more glucose and consumes more oxygen than any other organ in the body and generates an enormous amount of heat given its size relative to the total body mass. Brain temperatures are not a simple reflection of core body temperature; instead, they are higher than the body temperature and heterogeneous [for a review, see Wang et al. (54)]. Intracerebral temperatures vary across the dorsal–ventral axis. Brain temperatures are lowest in the cortex and highest in the midbrain core and ventral surface of the brain. The lower temperatures along the dorsal surface of the brain are due to greater radiation and convection of heat through the large surface of the cerebrum and relatively thin overlying skull and skin (54). It is interesting to note that the somatosensory and motor cortices of the cerebrum do not show a significant diurnal variation in the redistribution of CA. This is also true of the olfactory bulbs, although they have a high vascular density. The bulbs benefit from both the proximity to the skull as well as the continuous flow of air and blood to help regulate heat. These observations raise the possibility that temperature of the brain has a significant effect on clearance. Heat would affect the kinetics of diffusion in the parenchyma. Higher brain temperatures would enhance the movement of solute through the ISF toward and along the perivascular spaces lining blood vessels. The brain serves as a heat source and the blood as a heat sink. The temperature of the brain varies over the sleep–wake cycle of unrestrained rats. It is highest while rats are awake and active and lowest during sleep (55–57). Studies by Kiyatkin (58, 59) have shown increases in brain temperature in awake rats in response to multiple environmental stimuli and drugs of abuse. There is a thermal gradient in awake rats between the ventral striatum and systemic arterial blood that increases within seconds of tail pinch stimulation (60).

Certain brain areas have a high thermal sensitivity and are vulnerable to protracted hyperthermia. The excitability and intracellular calcium homeostasis of dopaminergic neurons in the substantia nigra compacta are sensitive to increases in brain temperature (61). The increased influx of calcium contributing to calcium overload may be a contributing factor to the degradation of dopaminergic neurons in this area associated with Parkinson’s. However, there are many factors contributing to the vulnerability and loss of dopaminergic neurons in Parkinson’s, such as oxidative stress by the normal catabolism of dopamine, glutamate toxicity, accumulation of inflammatory cytokines, and aggregation of α-synuclein protein (62). In a recently published review, Sundaram et al. (63) discussed the potential involvement in the glymphatic system, circadian regulation of the sleep–wake cycle, and clearance of α-synuclein with respect to Parkinson’s. Traumatic brain injury (TBI) can lead to the accumulation of α-synuclein aggregates in the midbrain dopaminergic system, contributing to the pathogenesis of Parkinson’s (64). In our own laboratory using multimodal MRI to follow the neuroradiological consequences of repetitive mild TBI, we found reduced functional coupling to the substantia nigra compacta with resting-state functional connectivity and alterations in gray matter microarchitecture 8 wk post head injury (65). In the present studies, we show a significant reduction in redistribution of CA from the substantia nigra compacta during the light phase of the diurnal cycle. While other aggregation-prone proteins, such as beta amyloid and tau, are cleared by the glymphatic system in models of Alzheimer’s disease and traumatic brain injury (66–68), α-synuclein clearance by the system has yet to be determined.

## Limitations and Speculation

This study was performed in awake rodents following the movement of tracer injected directly into the ventricular system. With the exception of parenchymal injections [see Carare et al. (69)], all other studies using fluorescent or paramagnetic tracers have been done under anesthesia following injection into the cisterna magna. As explained above, we chose the lateral ventricle because it is a logical site of injection given the natural movement of CSF along the anterior–posterior axis of the brain. Imaging animals during the scanning session while they are fully awake is something we pioneered and do routinely (70) because the results are easier to interpret and translate to the clinic. The inability to see movement following intraventricular injection under anesthesia (1) may be due to reduced heart rate and respiration, both of which are important in promoting convection along the ventricular system (36). The awake preparation is the most likely explanation for why we have higher redistribution of intraventricular CA from the glymphatic system than other MRI studies that used anesthesia (6, 27, 32–35). Anesthesia lowers brain temperature by as much as 3 to 4 °C below awake conditions (54). In fact, the anesthetized rat has a brain temperature that is lower than the core body temperature by 1 to 2 °C (71). The changes in brain metabolism, temperature, and reduction in blood flow with anesthesia should be considered when studying the diurnal movement of CSF and clearance through the perivascular system. It is unlikely that the use of anesthesia limits or blocks the movement of different tracers along the perivasculature and subarachnoid space given the many corroborating studies with different analytical methods and all infusing at an approximate rate of 1.6 µL/min. It is of interest to note that Bedussi et al. (49) achieved global labeling of the cisternal/ventricular/perivascular system with an infusion of 10 µL fluorescent probe at a rate of 0.34 µL/min over 30 min and gave evidence of fluorescent probe around and between smooth muscle pericytes and capillaries.

In these studies, the term “redistribution” is used to explain the influx and parenchymal distribution of intraventricular CA. The redistribution is lowest during the light phase when rats would normally be sleeping. Given what is known about glymphatic function, the low redistribution of intraventricular CA during phases of inactivity and sleep could be due to greater ISF efflux during this diurnal period. Conversely, the redistribution of intraventricular CA is highest during the diurnal period when the glymphatic system favors influx from the perivascular system to the parenchyma. If this is true, why are the whole-brain kinetics defining the influx of CA the same between light and dark phases in this study? Perhaps the movement of injected CA from CSF down the perivascular space of the macro- and microvasculature to start infiltration of probe into the abutting parenchyma may be similar across the light–dark cycle in the awake rat. However, the movement through the much smaller, highly tortuous interstitial space in the deeper parenchyma may be aided by influx during the dark phase and hindered by efflux during the light phase.

The higher density of medium and small arteries penetrating the midbrain also includes the accompanying perivascular spaces and underlying cisterns that together increase the clearance of heat and metabolites from the parenchyma of this region. However, the two processes of buffering heat and removing the waste of cerebral activity are out-of-phase. Why is the clearance through the glymphatic system less during the dark phase when brain activity is highest? This would seem counterintuitive. While only speculative, perhaps the hemodynamic changes of increased blood flow and transluminal pressures are limiting clearance by collapsing or reducing the perivascular space running along the microcirculation as reported by Bedussi et al. (49). Discussions and theoretical models for diffusion through the extracellular space from the parenchyma to and along the perivascular space for clearance do not include transluminal pressures (7, 72, 73) in the microcirculation. It was noted by Holter et al. (73) that the technology for measuring pressure gradients from blood vessels to parenchymal cells does not exist. The enhanced blood flow during the awake, active phase of the circadian cycle is necessary for moving blood gases and nutrients across the ISF–capillary interface and buffering temperature. The movement of nonpermeable metabolites and waste products like amyloid beta, tau, and alpha synuclein would be restricted during this period.

Awake animal imaging has its limitations and considerations. There was a limit set by our IACUC as to the time the rats could be restrained and imaged in the magnet. This limit was 2 h, preventing us from observing the full kinetics of influx and efflux over the 24-h light–dark cycle. Without EEG data, the state of rest and/or sleep is unknown. While the physiology and motion data would suggest the rats imaged during the light phase were awake, this cannot be certain. Regardless, as noted above, imaging rats during their light phase when they are resting or sleeping has elements of sleep deprivation, albeit for only 2 to 3 h. How much did this affect redistribution of CA? There is also the issue of stress associated with head restraint. While animals are acclimated to the imaging protocol reducing the autonomic signs of arousal and stress, they are unlikely to be stress-free. Activation of the hypothalamic–pituitary–adrenal axis during stress has a significant role in disrupting the sleep–wake cycle (74). While these imaging studies are acute, disrupting sleep and evoking stress for just a short period of time, the effects on CA redistribution are probably minimal, although it would be of interest to perform circadian imaging studies on rats housed for prolonged periods in stressful environments.

## Summary

Dynamic, contrast-enhanced MRI was used in awake rats to study the effect of the light–dark cycle on glymphatic redistribution of intraventricular CA across the whole brain. The findings are in agreement with previous studies showing clearance is highest during the diurnal cycle associated with rest and sleep, although it is unknown how much of the circadian variation was due to entrainment to the light–dark cycle versus the sleep–wake cycle and/or other physiological rhythms. The use of a 3D segmented and annotated MRI rat atlas enabled the identification of regional and brain-specific differences in redistribution of CA. Redistribution of intraventricular CA was highest along the dorsal cerebrum and lowest in the midbrain/pons and along the ventral surface of the brain. This heterogeneous distribution of CA influx and parenchymal distribution parallels the gradients and regional variations in brain temperatures reported in the literature for awake animals. These same brain areas of low redistribution of CA and high temperature have a high density of macrovasculature or small, medium, and large arteries and veins that may be an essential part of the organization of the perivascular system regulating brain temperature, blood gases, nutrients, metabolites, and waste products over the light–dark cycle.

## Supplementary Material

Supplementary File
