# Supplementary material for: Imaging the effect of the circadian light–dark cycle on the glymphatic system in awake rats
Source: Proc Natl Acad Sci U S A. 2019 Dec 17;117(1):668–76. doi: 10.1073/pnas.1914017117 (PMC6955326; doi:10.1073/pnas.1914017117)
Supplement: Supplementary File [file pnas.1914017117.sapp.pdf]

Supplementary Information Appendix File

Imaging the effect of the circadian light-dark cycle on the glymphatic system in awake rats

Xuezhu Cai[1], Ju Qiao[1], Praveen Kulkarni[1], Ian C Harding[2], Eno Ebong[3], Craig F Ferris[1,4]

[1]Center for Translational Neuroscience, [2]Department of Bioengineering, [3]Department of Chemical Engineering [4]Departments of Psychology and Pharmaceutical Sciences, Northeastern University, Boston, Massachusetts

Address all Correspondence to:
Craig Ferris, PhD, Director Center for Translational NeuroImaging
360 Huntington Ave, Boston Massachusetts, 02115
Email: c.ferris@northeastern.edu
Phone:508 259 4908

www.pnas.org/cgi/doi/10.1073/pnas.1914017116

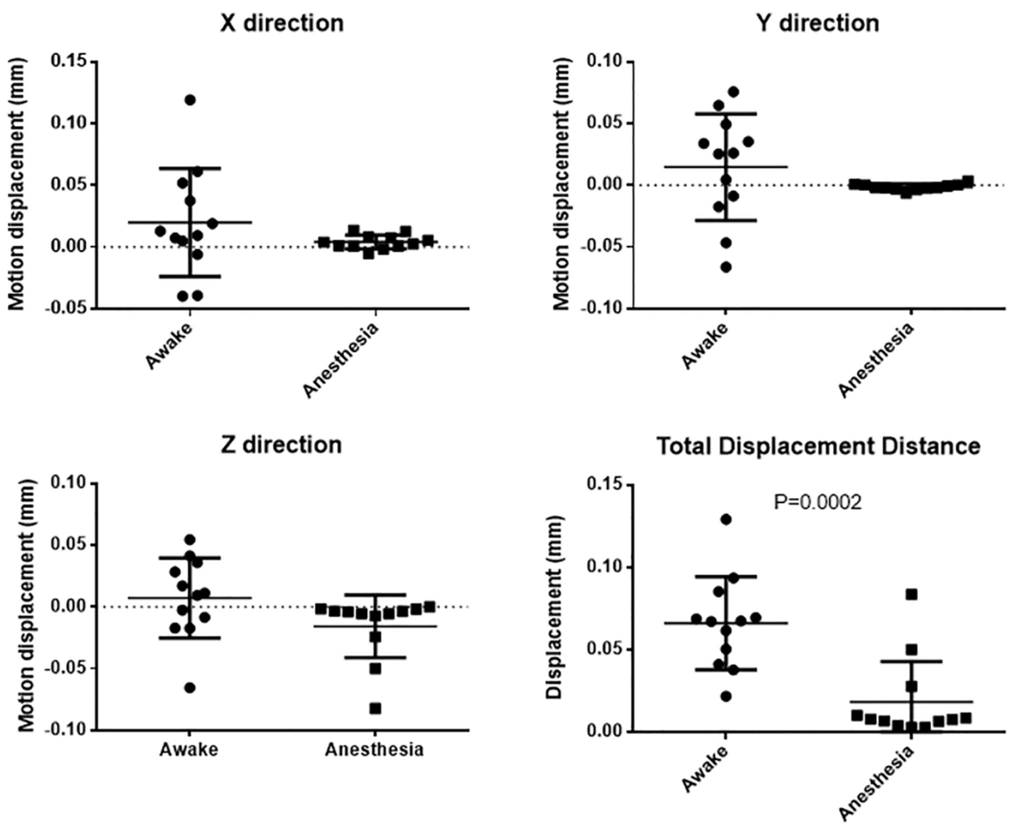

Figure 1S. Head motion in awake rats (n = 6) and anesthetized rats (n = 6) imaged during the light phase.

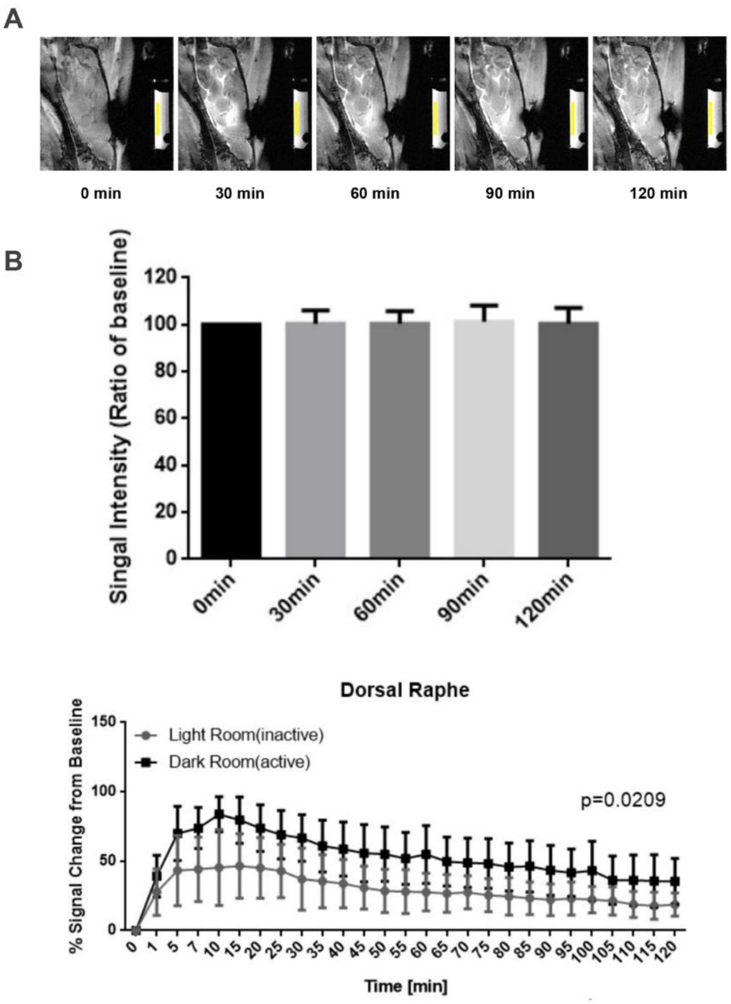

Figure 2S. Validation of image preprocessing with signal intensity re-scaling. (A) Representation of T1-weighted sagittal images with a colored ROI placed on the phantom adjacent to the brain at five different time points after intracerebroventricular injection of Gd-based contrast agent. (B) Quantification of re-scaled signal intensity extracted from the ROIs (n=6 per group). The bottom time-to-signal curve shows significance between the light-dark phases based on a two-way ANOVA followed by Sidak multiple correction.

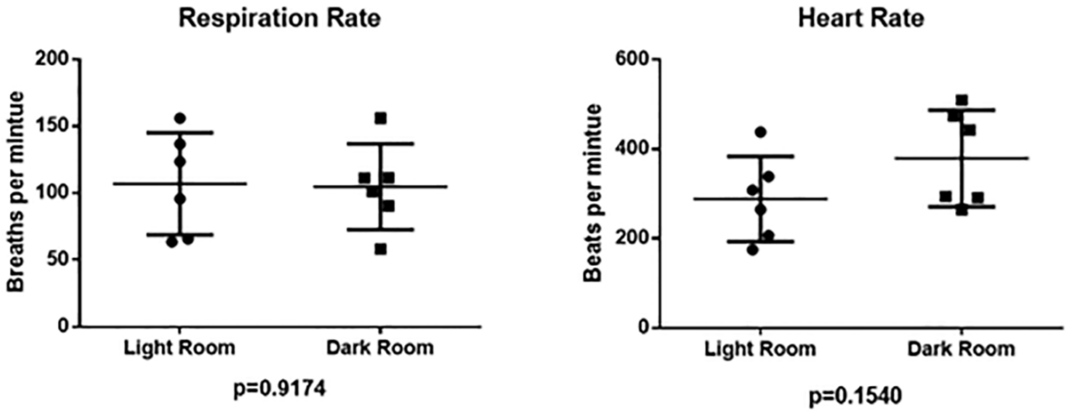

Figure 3S. Measures of respiration and heart rate during the scanning session in awake rats on reverse light-dark cycles.

Table 1. Comparison of parameters derived from regional fitted time-signal curves

Brain Region	Parameters	Light Room		Dark Room		P value
		Mean	SD	Mean	SD	
Suprachiasmatic Nucleus	AUC 0_60 *	2249	442.5	5077	2208	0.0117
	AUC 0_120 **	3230	742.6	8603	3877	0.0076
	Peak *	54.93	15.03	100.6	42.67	0.0329
	Peak Time	11.50	7.148	15.17	6.306	0.3683
Pedunculopontine Tegmental Area	AUC 0_60 *	861.5	319.1	1513	467.3	0.0181
	AUC 0_120 **	1798	715.5	3357	960.1	0.0097
	Peak **	17.65	6.861	33.70	9.570	0.0075
	Peak Time	48.17	10.13	60.67	14.17	0.1092
Supramammillary Nucleus	AUC 0_60 **	2094	228.7	4169	1388	0.0047
	AUC 0_120 **	3501	623.1	7468	2247	0.0019
	Peak **	44.32	9.508	80.92	25.56	0.0082
	Peak Time	18.00	10.43	23.00	13.97	0.4984
Sub Coeruleus Nucleus	AUC 0_60	489.1	295.1	821.9	230.6	0.0545
	AUC 0_120 *	1029	601.1	1949	563.4	0.0210
	Peak *	10.78	5.607	19.53	5.789	0.0240
	Peak Time	56.67	40.73	78.67	17.55	0.2522
Dorsal Raphe	AUC 0_60 *	2214	1111	3848	880.2	0.0180
	AUC 0_120 *	3548	1754	6349	1887	0.0238
	Peak *	47.28	21.58	79.34	13.42	0.0114
	Peak Time	17.50	21.87	12.17	8.542	0.5902
Pontine Nuclei	AUC 0_60 *	1141	538.4	2498	1138	0.0247
	AUC 0_120 **	2287	1086	4976	1737	0.0093
	Peak **	25.09	9.183	50.72	17.02	0.0088
	Peak Time	46.17	37.80	47.33	38.51	0.9588
Pontine Reticular Nucleus Oral	AUC 0_60	631.3	254.6	945.9	267.1	0.0633
	AUC 0_120 *	1545	543.4	2424	600.5	0.0240
	Peak *	15.55	4.960	25.69	6.340	0.0115
	Peak Time	87.17	21.67	85.50	18.84	0.8898
Entorhinal Cortex	AUC 0_60	922.1	338.4	1206	247.7	0.1278
	AUC 0_120 *	2109	561.4	2942	461.5	0.0185
	Peak *	22.06	5.407	30.65	4.657	0.0145
	Peak Time	73.17	28.05	83.00	23.07	0.5222
Hippocampus	AUC 0_60	1191	353.9	1646	414.9	0.0683
	AUC 0_120 *	2595	757.5	3625	625.7	0.0302
	Peak *	25.02	6.977	36.00	6.275	0.0168
	Peak Time	56.83	14.58	62.00	15.50	0.5653
Midbrain	AUC 0_60 *	1127	418.1	1743	571.9	0.0590
	AUC 0_120 *	2340	857.1	3819	1027	0.0220
	Peak *	23.55	8.071	38.30	10.20	0.0195
	Peak Time	46.17	28.14	59.83	12.67	0.3035

Striatum	AUC 0_60 *	897.2	393.3	1439	381.6	0.0361
	AUC 0_120	2028	836.3	3094	835.0	0.0516
	Peak	19.78	7.706	29.92	8.461	0.0552
	Peak Time	70.17	33.09	70.33	43.65	0.9942
Cerebellum	AUC 0_60	631.2	253.5	880.6	380.2	0.2108
	AUC 0_120	1248	503.1	1914	755.9	0.1028
	Peak	12.66	4.886	18.59	7.192	0.1260
	Peak Time	42.17	18.10	69.50	28.28	0.0741
Somatosensory Cortex	AUC 0_60	855.9	318.7	850.4	272.6	0.9776
	AUC 0_120	2138	714.3	2279	670.1	0.7568
	Peak	22.24	6.567	25.00	6.897	0.5348
	Peak Time	106.2	21.84	117.8	3.033	0.2734
Somatosensory Cortex Barrel Field	AUC 0_60	717.8	326.6	877	259.9	0.3724
	AUC 0_120	1655	804.8	2060	646.8	0.3595
	Peak	16.23	7.779	21.85	5.539	0.1801
	Peak Time	87.17	34.08	100.3	34.93	0.5236
Substantia Nigra	AUC 0_60 **	730.9	227.9	1667	498.1	0.0019
	AUC 0_120 ***	1628	513.8	3782	842.8	0.0003
	Peak ***	16.88	4.957	38.29	7.117	0.0001
	Peak Time	60.00	25.31	80.33	30.65	0.2387
Cerebellar Lobule	AUC 0_60	644.7	302.6	878.6	475.6	0.3334
	AUC 0_120	1289	633.2	1881	929.7	0.2268
	Peak	12.87	5.899	18.32	9.381	0.2556
	Peak Time	49.50	37.18	75.67	35.51	0.2409
Reticular Nucleus Midbrain	AUC 0_60 **	801.2	264.6	1401	254.5	0.0025
	AUC 0_120 **	1828	630.9	3228	462.7	0.0014
	Peak **	17.57	6.437	32.06	4.742	0.0013
	Peak Time	70.17	30.75	77.50	23.86	0.6543
Olfactory Bulb	AUC 0_60	880.9	665.9	1224	659.7	0.3914
	AUC 0_120	1867	991.9	2911	1145	0.1752
	Peak	20.31	11.57	30.50	14.78	0.2130
	Peak Time	67.33	40.50	72.33	26.65	0.8057

$p < 0.05$ was considered statistically significant.
